# Spatial Optimization Methods for Malaria Risk Mapping in Sub‐Saharan African Cities Using Demographic and Health Surveys

**DOI:** 10.1029/2023GH000787

**Published:** 2023-10-06

**Authors:** Camille Morlighem, Celia Chaiban, Stefanos Georganos, Oscar Brousse, Nicole P. M. van Lipzig, Eléonore Wolff, Sébastien Dujardin, Catherine Linard

**Affiliations:** ^1^ Department of Geography University of Namur Namur Belgium; ^2^ ILEE University of Namur Namur Belgium; ^3^ Geomatics Unit Department of Environmental and Life Sciences Karlstad University Karlstad Sweden; ^4^ Institute of Environmental Design and Engineering University College London London UK; ^5^ Department of Earth and Environmental Sciences Katholieke Universiteit Leuven Leuven Belgium; ^6^ Department of Geoscience, Environment & Society Université Libre de Bruxelles Brussels Belgium; ^7^ NARILIS University of Namur Namur Belgium

**Keywords:** urban malaria, sub‐Saharan Africa, DHS, remote sensing, random forest

## Abstract

Vector‐borne diseases, such as malaria, are affected by the rapid urban growth and climate change in sub‐Saharan Africa (SSA). In this context, intra‐urban malaria risk maps act as a key decision‐making tool for targeting malaria control interventions, especially in resource‐limited settings. The Demographic and Health Surveys (DHS) provide a consistent malaria data source for mapping malaria risk at the national scale, but their use is limited at the intra‐urban scale because survey cluster coordinates are randomly displaced for ethical reasons. In this research, we focus on predicting intra‐urban malaria risk in SSA cities—Dakar, Dar es Salaam, Kampala and Ouagadougou—and investigate the use of spatial optimization methods to overcome the effect of DHS spatial displacement. We modeled malaria risk using a random forest regressor and remotely sensed covariates depicting the urban climate, the land cover and the land use, and we tested several spatial optimization approaches. The use of spatial optimization mitigated the effects of DHS spatial displacement on predictive performance. However, this comes at a higher computational cost, and the percentage of variance explained in our models remained low (around 30%–40%), which suggests that these methods cannot entirely overcome the limited quality of epidemiological data. Building on our results, we highlight potential adaptations to the DHS sampling strategy that would make them more reliable for predicting malaria risk at the intra‐urban scale.

## Key Points

- We tested spatial optimization approaches to overcome the effect of cluster spatial displacement in Demographic and Health Surveys (DHS)
- Spatial optimization reduced the effect of displacement, but the percentage of variance explained in malaria models remained low
- We proposed potential adaptations to the DHS sampling strategy to better support the study of malaria risk at the intra‐urban scale

## 1 Introduction

Uncontrolled urbanization rates have transformed the cityscape of sub‐Saharan African (SSA) cities and their suburbs, bringing with them changes in the socioeconomic and demographic contexts (Georganos et al., 2020; Robert et al., 2003). More than 40% of SSA population already lives in cities, and this figure is expected to reach 58% by 2050 (United Nations et al., 2019). At the same time, SSA is also impacted by the current climate change, where climate projections foresee a warming trend with increased temperatures and disrupted rainfall (Serdeczny et al., 2017). This rapid urbanization and global climate change are likely to influence the epidemiology of vector‐borne diseases such as malaria (Kabaria et al., 2016; Serdeczny et al., 2017). In SSA, malaria‐related deaths still represent more than 95% of the global burden and almost all (i.e., 99.7%) are caused by the *Plasmodium falciparum* (*Pf*) parasite (Sinka et al., 2012; World Health Organization, 2021). In this context of change, there is an urgent need for a better understanding of intra‐urban malaria risk in SSA.

Malaria has often been studied in rural settings, where it is significantly more endemic than in urban areas (Kabaria et al., 2016; Tatem et al., 2013). However, urban environments are associated with higher population densities, which means that more people are exposed to malaria transmission risk (Doumbe‐Belisse et al., 2021). Besides, cities are highly heterogeneous environments, where malaria risk is also likely to be heterogeneous (Boyce et al., 2019; Machault et al., 2010). This means that rural malaria models are not easily applicable to urban areas. In recent years, research initiatives have investigated intra‐urban malaria risk in relation to driving factors such as climate (Brousse et al., 2020b, 2020c; Morlighem et al., 2022), land use and land cover (Georganos et al., 2020; Kabaria et al., 2016; Morlighem et al., 2022) and socioeconomic variables (Georganos et al., 2020; Morlighem et al., 2022). In this previous work, high‐risk urban areas were associated with informal settlements built on lowlands, proximity to wetlands and urban agriculture.

Studying intra‐urban malaria requires high‐resolution data that capture the spatio‐temporal heterogeneity of intra‐urban epidemiology and its local determinants (i.e., malaria driving factors). The availability of epidemiological data has increased considerably in recent years, with various types of surveys being conducted as part of scientific research or larger scale studies. Common data sources are the Demographic and Health Surveys (DHS) and the Malaria Indicator Surveys (MIS) of the same DHS program (The DHS Program, 2022)—from this point on, we use the term “DHS” to refer to both DHS and MIS to avoid repetition. These are nationally sampled, cross‐sectional, geolocated surveys conducted periodically in more than 90 developing countries (Corsi et al., 2012; Georganos et al., 2019). Besides, since the Malaria Atlas Project in the early 2000s (Hay & Snow, 2006; Malaria Atlas Project, 2022), remote sensing and geographic information systems have been increasingly used for low‐resolution, large‐scale mapping of malaria risk (Adigun et al., 2015; Riedel et al., 2010) and, more recently, for city‐specific, high‐resolution mapping (Georganos, 2020; Morlighem et al., 2022). This has led to the production of fine‐scale maps of the environmental and socioeconomic factors that influence intra‐urban malaria risk (e.g., Kabaria et al. (2016) and Rogers et al. (2002)). In recent years, several studies have combined DHS and remote sensing to map malaria risk at the national or sub‐national level (Adigun et al., 2015; Ejigu, 2020; Giardina et al., 2012; Riedel et al., 2010; Ssempiira et al., 2017).

Nevertheless, a well‐known issue with DHS epidemiological data is that survey cluster coordinates are randomly displaced by up to 2 km in urban areas (5 km in rural areas) before publication to protect the privacy of survey participants (Burgert et al., 2013; Corsi et al., 2012; Georganos et al., 2019; Ozodiegwu et al., 2021). Although it has little impact at the national scale, at the intra‐urban scale it reduces the spatial accuracy of the DHS indicators and may in turn affect the spatial accuracy and reliability of intra‐urban malaria risk maps that rely on DHS (Ozodiegwu et al., 2021). Some spatial optimization methods have recently been proposed to adjust for this displacement effect when modeling the DHS wealth index at the intra‐urban scale (Georganos et al., 2019). These are based on the duplication of the published DHS cluster coordinates in the four cardinal directions (N, S, E, W) to enrich or refine the contextual spatial feature extraction (Georganos et al., 2019). In this paper, we further investigate these methods to map intra‐urban malaria risk with DHS.

The main objective of this study is to test the potential of spatial optimization methods to overcome the loss of spatial accuracy due to survey cluster displacement when modeling and predicting intra‐urban malaria risk in SSA using DHS. We focus on four SSA cities for which data are available: Dakar (Senegal), Dar es Salaam (Tanzania), Kampala (Uganda) and Ouagadougou (Burkina Faso). The main modeling workflow involves a popular machine learning algorithm, namely random forest (RF), and multi‐resolution remotely sensed variables representing the urban climate, the land use and the land cover. In addition, we test the spatial optimization methods on non‐DHS data for which we simulated a spatial displacement according to the DHS procedure. Building on our results, we discuss potential adaptations of the DHS sampling strategy so that they can better support intra‐urban malaria risk mapping.

## 2 Materials and Methods

### 2.1 *Plasmodium falciparum* Prevalence Data

*Plasmodium falciparum* (*Pf*) prevalence data were extracted from an open source malaria database (Snow et al., 2017a), which contains survey data compiled from various sources such as DHS or scientific research from 1900 to 2015 (Snow et al., 2017b). Data are available as geolocated data points accompanied with the timestamp of the survey, the age range of the population sampled, the size of the sample and the *Pf* prevalence. *Pf* prevalence is measured as the *Pf* Parasite Rate standardized to the 2 to 10 age range (*Pf*PR_2–10_). The *Pf*PR is the proportion of the surveyed human population with detectable *Pf* parasites in their peripheral blood (Smith et al., 2007). As this measure varies across age groups with human immunity, the metric is standardized over a child age range following Smith et al. (2007) to ensure comparability between malaria surveys conducted on different age samples (Smith et al., 2007).

This database has been used in previous work to predict malaria risk at the intra‐urban scale in several SSA cities (including Dar es Salaam, Kampala and Dakar) (Brousse et al., 2020b; Georganos et al., 2020; Kabaria et al., 2016; Morlighem et al., 2022), but DHS were usually filtered out of the database and malaria models relied only on non‐displaced survey data, which drastically reduced the number of available survey points. Here, we selected all types of surveys conducted between 2005 and 2015, assuming stable malaria prevalence over this period, as a compromise between temporal consistency and the number of available surveys. We did not consider surveys in which participants were older than 16 years to ensure that *Pf*PR_2–10_ estimates are not affected by the increased mobility of older individuals (Brousse et al., 2020b). The four cities selected are those with the highest number of survey data points after applying these selection criteria. Table 1 shows the number of surveys available for each city, together with their mean *Pf*PR_2–10_ and standard deviation. Despite the variety of survey types, many of the surveys are DHS, in which the urban cluster coordinates are randomly displaced within 2 km buffers to protect the privacy of the respondents. The only restriction on displacement is that data points must remain within the boundaries of the administrative unit (usually the second administrative level) to which they belong, and this only applies to surveys conducted after 2008 (Burgert et al., 2013). Given this diversity in survey types, the spatial accuracy of the data points varies within and between cities, for example, Dakar and Ouagadougou (see Table 1).

**Table 1:** Surveys Meeting the Selection Criteria, *Pf*PR_2–10_ Statistics and Demographic and Health Surveys for Each City

	Selected surveys	Mean *Pf*PR_2–10_ ± SD	DHS
Dakar	122	1.4 ± 3.0	88 (72.1%)
Dar es Salaam	172	5.2 ± 7.5	82 (47.7%)
Kampala	77	5.0 ± 6.9	38 (49.4%)
Ouagadougou	52	15.2 ± 23.4	52 (100%)

*Note*. The selection criteria are all surveys conducted over 2005–2015 and including only participants aged less than 16. SD stands for “standard deviation.”

### 2.2 Remotely Sensed Predictors

In this study, we conjointly used as predictors data sets that have separately been shown to be useful for mapping intra‐urban malaria risk in SSA cities. We gathered the Land Cover and Land Use (LCLU) data sets used in Georganos et al. (2020) to map *Pf*PR_2–10_ in Kampala and Dar es Salaam, and the Local Climate Zones (LCZ) and ancillary variables used in Brousse et al. (2020b) to map *Pf*PR_2–10_ in nine SSA cities. On top of these data sets, we assembled climatic variables from different data sources. These data sets are detailed in the following sections.

#### 2.2.1 Land Cover and Land Use (LCLU)

The LC maps (50 cm resolution) were generated following the processing toolchain of Grippa et al. (2017), which performs LC classification from satellite imagery. The LC classification is implemented by a combination of Computer Assisted Photo Interpretation, Geographic Object Based Image Analysis and machine learning algorithms. The satellite images on which the classification is based are Pleiades images of Dar es Salaam (acquired in March and January 2016 and July 2018), Kampala (acquired in February 2013) and Dakar (acquired in July 2015), and WorldView3 VHR images of Ouagadougou (acquired in October 2015).

Following Grippa et al. (2018), LU maps (20 m resolution) were created from the aforementioned LC maps. Spatial metrics derived from the LC maps were used to perform LU classification of street blocks reconstructed from OpenStreetMap parcels and street networks. All LCLU maps are freely available from Zenodo scientific repositories (Grippa & Georganos, 2018a, 2018b, 2018c, 2019; Georganos, 2020; Georganos & Grippa, 2020a, 2020b) and have been used in previous research to map intra‐urban malaria risk in Kampala and Dar es Salaam (Georganos et al., 2020; Morlighem et al., 2022). The LC and LU classes are detailed in Table 2.

**Table 2:** Remotely Sensed Predictive Variables of *Pf*PR_2–10_

Variables	Spatial resolution	Type of extraction
Land cover and land use		
Low vegetation (humid, riparian, grasses, bushes) (LC)	50 cm	Proportion
Tall vegetation (LC)	50 cm	Proportion
Water (LC)	50 cm	Proportion
Building (LC)	50 cm	Proportion
Bare ground (LC)	50 cm	Proportion
Planned settlements (LU)	20 m	Proportion
Informal settlements (LU)	20 m	Proportion
Wetlands, streams, marshes, rivers (mixed class) (LU)	20 m	Proportion
Non‐residential built‐up (ACS: Administrative Commercial Service) (LU)	20 m	Proportion
Local climate zones		
Compact built areas	100 m	Proportion
Open built areas	100 m	Proportion
Water bodies	100 m	Proportion
Lowlands	100 m	Proportion
Trees	100 m	Proportion
Industrial areas	100 m	Proportion
Climatic variables		
Maximum day LST	1 km	Direct extraction
Minimum day LST	1 km	Direct extraction
Mean day LST	1 km	Direct extraction
Maximum night LST	1 km	Direct extraction
Minimum night LST	1 km	Direct extraction
Mean night LST	1 km	Direct extraction
Mean daily LST range	1 km	Direct extraction
Maximum precipitation	1 km	Direct extraction
Minimum precipitation	1 km	Direct extraction
Mean precipitation	1 km	Direct extraction
Maximum windspeed	1 km	Direct extraction
Minimum windspeed	1 km	Direct extraction
Mean windspeed	1 km	Direct extraction
Ancillary variables		
Mean NDVI	100 m	Average
Mean NDWI	100 m	Average
Elevation	30 m	Average

#### 2.2.2 Local Climate Zones (LCZ)

LCZ are meaningful LCLU classes that describe the urban climate, where each class represents a specific urban typology with its inherent climate (Brousse et al., 2020b). The LCZ maps (100 m resolution) used in this study are derived from an RF classification applied to Landsat, USGS and Sentinel imagery from 2017 to 2019 (Brousse et al., 2020b) and are freely available from the LCZ Generator (Demuzere et al., 2021). Training areas used in the RF classification originated from a mapathon organized in November 2019 and have served the mapping of intra‐urban malaria epidemiology for nine SSA cities (Brousse et al., 2020a, 2020b).

Table 2 shows the LCZ classes used in this study.

#### 2.2.3 Climatic Variables

We gathered meteorological satellite observations from MODIS data collections (products MYD11A1 v006 and MOD11A1 v006) at 1 km resolution (Wan et al., 2015a, 2015b) to account for intra‐urban climatic variations. From the daily averages of TERRA and AQUA day and night Land Surface Temperatures (LST), we extracted the maximum, minimum and mean year‐monthly day and night LST and the mean daily LST range (calculated as the difference between the maximum and minimum daily LST) over a 13‐year period (2005–2017). In addition, we used the maximum, minimum and mean year‐monthly precipitation and windspeed over 1970–2000 from WorldClim climate data (1 km resolution) (Fick & Hijmans, 2017).

#### 2.2.4 Ancillary Variables

Finally, following Brousse et al. (2020b), we included additional variables for which the relationship with malaria prevalence has often been highlighted (Kabaria et al., 2016; Sewe et al., 2016): (a) the averaged Normalized Difference Vegetation and Water Indices (NDVI and NDWI) over period 2005–2019 derived from Landsat 5 and 8 satellite imagery (100 m resolution) and (b) a digital elevation model of 2000 from the Shuttle Radar Topography Mission (SRTM) (30 m resolution) (Rodríguez et al., 2006).

Table 2 provides a summary of all the predictor variables investigated in this study.

#### 2.2.5 Covariate Extraction

For 1 km resolution variables, that is, climatic variables, covariate values were extracted directly at the malaria survey coordinates. For all LC, LU and LCZ variables, we calculated the proportion of each class contained within 1 km buffers around the geolocated surveys. Finally, we used 1 km buffers to extract averaged NDVI, NDWI and SRTM values. In this way, all covariates were aggregated at the same 1 km resolution.

### 2.3 Modeling and Predicting *Pf*PR_2–10_

#### 2.3.1 Random Forest Modeling

We used an RF regressor to model *Pf*PR_2–10_ from the set of remotely sensed covariates. RF is a robust algorithm for dealing with multicollinearity and allows for non‐linear relationships between the covariates and the dependent variable (Breiman, 2001). RF is an ensemble method based on decision trees and uses bagging to reduce overfitting (Breiman, 2001). As spatial autocorrelation tends to bias variable importance in RF modeling (Meyer et al., 2019), we used five‐repeated five‐fold spatial cross‐validation (25 RF models in total), which means that 80% of the data was used for training and 20% for testing (Lovelace et al., 2019). Hyperparameter tuning was performed by further dividing each fold into five sub‐folds and fitting 50 RF models to define optimal values for (a) the number of covariates used at each node split (ranging from one to the number of covariates decremented by one), (b) the minimum number of observations per terminal node (ranging from one to 10), and (c) the fraction of observations used per decision tree (ranging from 0.2 to 0.9) (Lovelace et al., 2019).

To identify the most relevant covariates for predicting *Pf*PR_2–10_, we implemented a feature selection method called recursive feature elimination (RFE) (Gregorutti et al., 2017). This method works by iteratively removing the least important covariate from the set of predictors until the predictive performance is the highest (Gregorutti et al., 2017). Here, covariate importance is measured as the standardized increase in the averaged Out of Bag (OOB) error of the RF models after random permutation of the covariate values. The most important covariates produce the largest increases in OOB error when permuted (Breiman, 2001). To measure the predictive performance at each iteration of the RFE, we used three goodness‐of‐fit (GoF) indices (calculated on the test set): the root mean square error (RMSE), the mean absolute error (MAE) and the coefficient of determination (*R* ^2^), which is calculated using Equation 1, where *n* is the number of observations, *y*_*i*_ is the value of observation *i*, ${\hat{y}}_{i}$ is its predicted value and $\bar{y}$ is the mean of all observed values.

(1) $$R^{2}=1-\frac{\sum_{i=1}^{n}\left(y_{i}-{\hat{y}}_{i}\right)²}{\sum_{i=1}^{n}\left(y_{i}-\bar{y}\right)²}$$

#### 2.3.2 Spatial Optimization Methods

**Figure 1:**
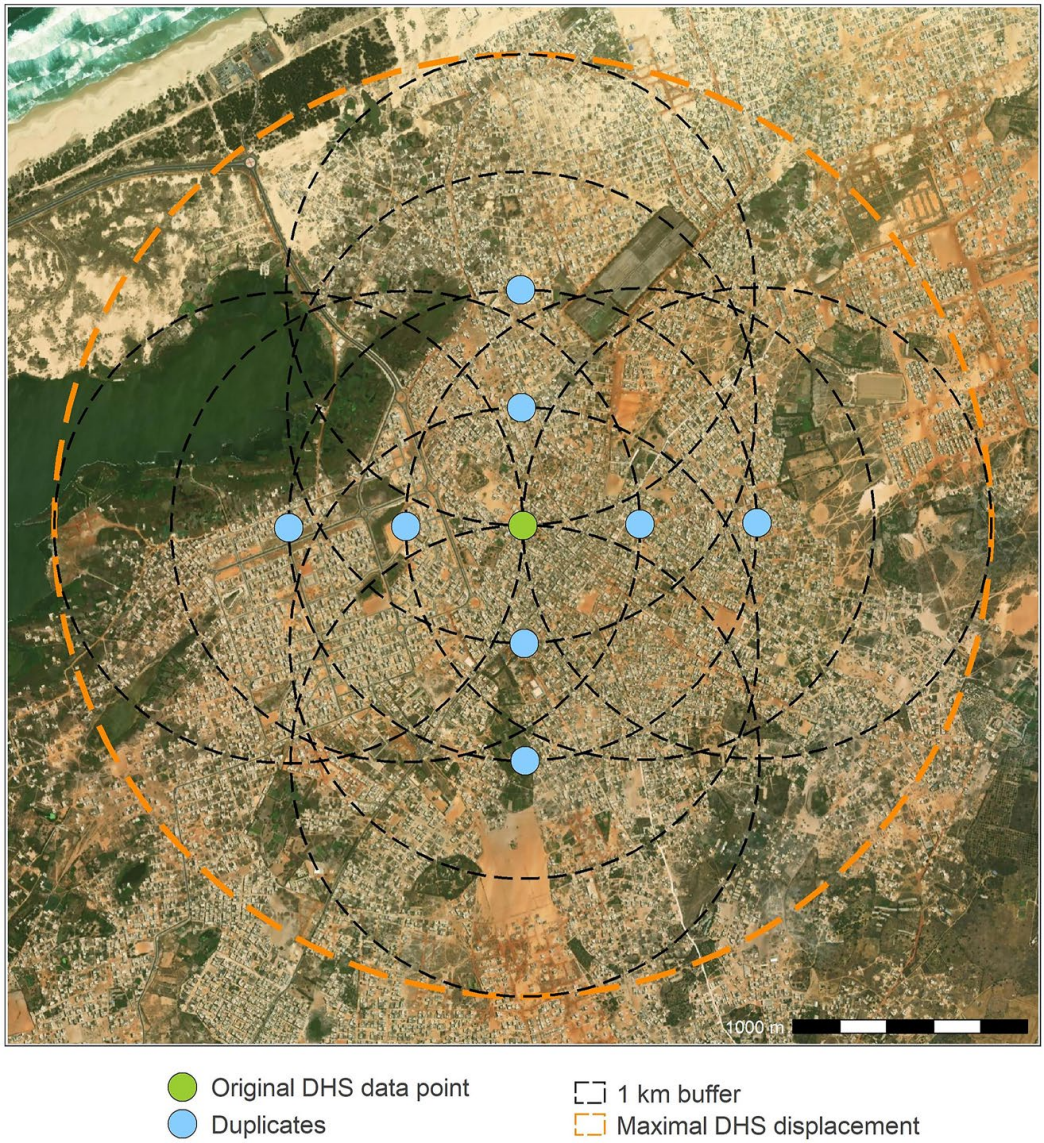
Example of duplication of a data point from the Demographic and Health Surveys in Dakar. The data point is duplicated in the four cardinal directions (N, S, E, W) at 500 and 1,000 m. The figure is adapted from Georganos et al. (2019) and the background map is Esri World Imagery (Esri et al., 2021).

As mentioned in Section 2.1, many malaria surveys are DHS. As a result, the displacement of survey cluster coordinates is likely to reduce the predictive performance of RF models. The displacement occurs within a 2 km radius, and variables describing the cityscape, such as LCLU, can change drastically within this range in urban settings (Gething et al., 2015). To mitigate the effects of dislocation, we investigated two spatial optimization methods recently proposed by Georganos et al. (2019) to overcome the effects of survey cluster displacement when modeling the DHS wealth index. The first method (M1) consists of enriching the size of the training and test sets by duplicating the DHS data points in the four cardinal directions (N, S, E, W) at 500 m and 1,000 m—resulting in a ninefold increase in the number of DHS data points—as shown in Figure 1. Covariates are extracted and RF modeling is implemented along with RFE using the original DHS data points and the newly created duplicates. With this method, we assumed that at least 50% of the DHS clusters are displaced within a 1 km buffer, following Georganos et al. (2019). In addition, when this technique is combined with covariate extraction in 1 km buffers around the duplicates, the maximum 2 km range of DHS displacement is almost completely covered, as can be seen in Figure 1.

The second method (M2) involves data refinement rather than enrichment. Instead of using all nine potential locations (i.e., eight duplicates and one original data point) for each DHS data point, one of them is randomly selected—leaving the total number of data points unchanged—and the final random selection is used for covariate extraction and RF modeling. This process is repeated over 1,000 iterations, and we finally use the data points of the best performing iteration (as measured by the GoF indices, i.e., RMSE, MAE and *R* ^2^) to implement the RFE.

Eventually, we used M1, M2 and M0, that is, the base RF modeling method implemented without any spatial optimization, to model malaria risk in the four cities of interest, and compared the performance of these methods using the GoF indices. In this study, we retained all types of surveys, allowing us to test spatial optimization methods for different case studies, with varying proportions of DHS, ranging from around 50% (Kampala and Dar es Salaam) to 100% (Ouagadougou) (see Table 1). Using the best performing method among M0, M1 and M2, we predicted *Pf*PR_2–10_ in the four cities of interest on a 1 km resolution grid. The final predictive maps are obtained by averaging the predictions of the 25 RF models built in spatial cross‐validation.

#### 2.3.3 Simulation Study

Following Macharia et al. (2022), we conducted a simulation study to also test the two above‐mentioned spatial optimization methods on simulated data. This simulation study focuses on the city with the highest number of non‐DHS surveys, that is, Dar es Salaam, resulting in 90 surveys (see Table 1). The simulation was implemented through the following steps:

1. Non‐DHS data were split into a training set (77%) and a test set (33%), and the training set was spatially displaced according to the DHS displacement method (including the constraint that data points must stay within the same level 2 administrative area), see Burgert et al. (2013) for more details on the exact procedure.
2. Three models were fitted to the displaced training set: the base RF modeling method implemented without using any spatial optimization (M0) and the two spatial optimization methods (M1 and M2). The administrative area constraint was considered in the implementation of M1 and M2 by only creating duplicates in the same level 2 administrative area as the (displaced) data‐point. In addition to M0, M1 and M2, an RF model with an RFE was fitted to the original training set, that is, before displacement, and is referred to as the true model (TM). Note that all RF models here were built in random cross‐validation as we were not interested in variable importance and for the sake of computational efficiency.
3. All four models (M0, M1, M2 and TM) were used for predictions on the non‐displaced test set, and GoF indices (RMSE, MAE and *R* ^2^) were calculated to compare predicted and observed values.

We repeated this process 10 times, including the separation into training and test sets. Predictive performance was summarized over these 10 iterations, using averaged GoF indices, and used to compare M0, M1, M2, and TM.

## 3 Results

### 3.1 Performance of *Pf*PR_2–10_ Models

**Figure 2:**
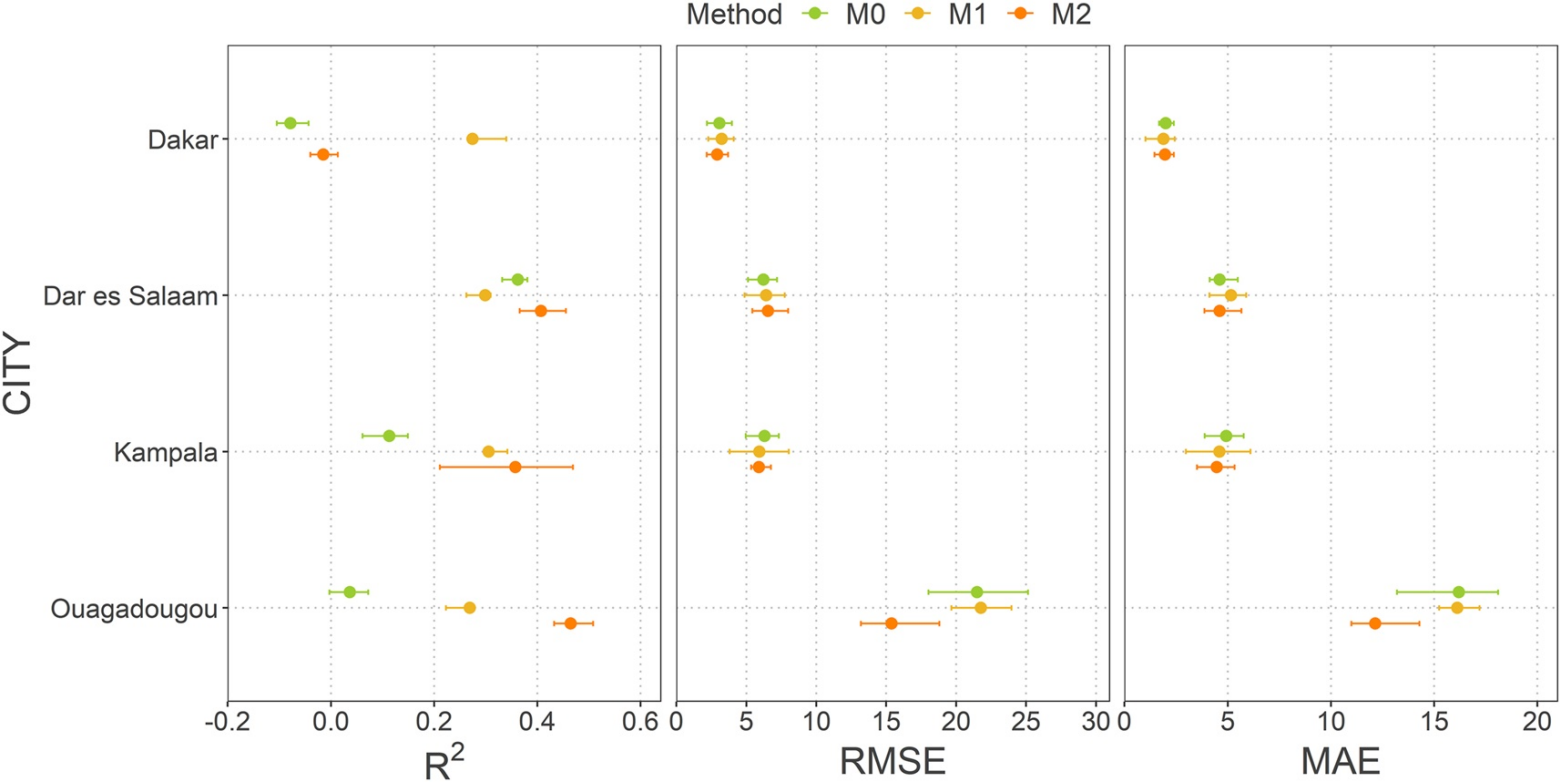
Average *R* ^2^, root mean square error (RMSE) and mean absolute error (MAE) scores obtained with M0, M1 and M2. A recursive feature elimination (RFE) was implemented for each of M0, M1 and M2, and the values shown correspond to the RFE iteration with the highest predictive performance. The thick dots are the averaged metrics (*R* ^2^, RMSE and MAE) over 25 random forest models built in spatial cross‐validation, and the error bars represent the interquartile range calculated over these 25 models.

The GoF indices (RMSE, MAE and *R* ^2^) for the different methods implemented (M0, M1 and M2) and for each city are shown in Figure 2 and Table 3. An RFE was implemented for each of these methods and the values displayed correspond to the best performing models after removing non‐significant covariates.

**Table 3:** Average *R* ^2^, Root Mean Square Error (RMSE) and Mean Absolute Error (MAE) Scores Obtained With M0, M1 and M2

	Method	*R* ^2^	RMSE	MAE
Dakar	M0	−0.08	3.08	2.00
	M1	0.27	3.23	1.88
	M2	−0.01	2.91	1.97
Dar es Salaam	M0	0.36	6.21	4.61
	M1	0.30	6.41	5.16
	M2	0.41	6.54	4.61
Kampala	M0	0.11	6.31	4.93
	M1	0.31	5.94	4.60
	M2	0.36	5.89	4.47
Ouagadougou	M0	0.04	21.49	16.20
	M1	0.27	21.76	16.12
	M2	0.46	15.39	12.15

*Note*. The scores are averaged over 25 RF models (five‐repeated five‐fold spatial cross‐validation) for each city.

In Dakar, M1 performed better than the other two methods in terms of *R* ^2^ and MAE, with an *R* ^2^ of 0.27 and an MAE of 1.88. In Kampala, M2 was the best performing method for all three GoF indices, with an average *R* ^2^ of 0.36, RMSE of 5.89 and MAE of 4.47. M1 also outperformed M0 on all three GoF indices. In Ouagadougou, M2 was also the best performing method according to all three GoF indices, with an average *R* ^2^ of 0.46, an average RMSE of 15.39 and an average MAE of 12.15. M1 also showed a higher performance than M0 in terms of *R* ^2^ and MAE. Finally, in Dar es Salaam, M2 performed best in terms of *R* ^2^ and MAE, with an average *R* ^2^ of 0.41 and an average MAE of 4.61. However, unlike all other cities, M1 did not outperform M0 in any of the GoF indices.

For all cities, regardless of the proportion of DHS data points used to build the models, the best performing method is either M1 or M2—i.e. M1 for Dakar and M2 for Dar es Salaam, Kampala and Ouagadougou—with the models from worst to best performing (in terms of *R* ^2^) being those of Dakar (0.27), Kampala (0.36), Dar es Salaam (0.41) and Ouagadougou (0.46). These differences in *R* ^2^ could be explained by several factors, such as the number of malaria surveys available for each city, but also the regional level of malaria endemicity, the cityscape heterogeneity within cities, and the extent to which the covariates used are good predictors of intra‐urban malaria risk in each city. RMSE and MAE values should not be compared between cities, as they depend on the number of surveys and the distribution of *Pf*PR_2–10_ values in each city. The wider distribution of *Pf*PR_2–10_ values in Ouagadougou (Table 1) could explain the higher RMSE and MAE values (15.39 and 12.15) compared to the other three cities, which show less variability in their *Pf*PR_2–10_ values.

### 3.2 Importance of Covariates

**Figure 3:**
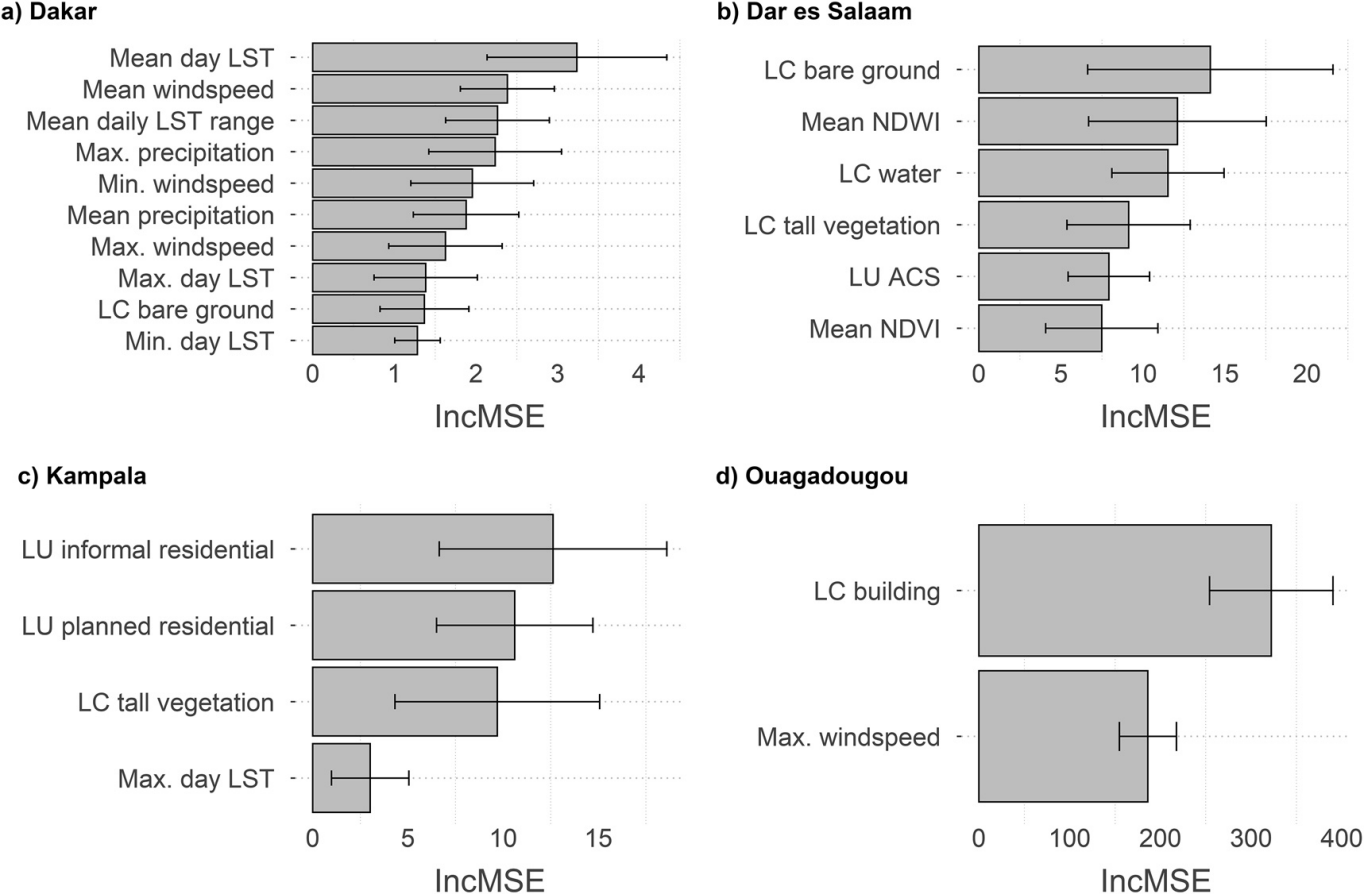
Covariate importance for each city of interest. Covariate importance is computed over 25 random forest (RF) models (five‐repeated five‐fold spatial cross‐validation) using recursive feature elimination together with the best performing method for each city, that is, M1 for Dakar and M2 for Dar es Salaam, Kampala and Ouagadougou. The importance of a covariate is the increase in the Out of Bag error (measured as a mean squared error) after applying a random permutation of the covariate values. The error bars represent the standard deviation calculated across 25 RF models.

Feature selection was implemented through RFE. Figure 3 shows the covariates included in the best models (i.e., M1 for Dakar and M2 for Dar es Salaam, Kampala and Ouagadougou) and their importance. The RFE selected 10 important covariates for Dakar, all of which are climatic (mean, maximum and minimum day LST, mean, maximum and minimum windspeed, mean daily LST range and mean and maximum precipitation) except for one which is part of the LCLU data set (the proportion of bare ground) (Figure 3a). In Dar es Salaam, the RF models included four LCLU covariates (the proportion of bare ground, water, tall vegetation and ACS) and two ancillary covariates (the mean NDWI and NDVI) (Figure 3b). In Kampala, the main predictors of *Pf*PR_2–10_ were three LCLU covariates (the proportion of informal settlements, planned settlements and tall vegetation) and one climatic covariate (the maximum day LST) (Figure 3c). Finally, the RF models in Ouagadougou included only two covariates: the proportion of buildings and the maximum windspeed (Figure 3d).

In short, these results show the importance of climatic variables in modeling and predicting *Pf*PR_2–10_ in Dakar, Ouagadougou and Kampala. In Dar es Salaam, most of the important covariates were instead LCLU covariates. LCLU covariates were also important for modeling *Pf*PR_2–10_ in the other cities, as there is at least one important LCLU covariate in the model of each city, and for all cities except Dakar, the most important covariate was either an LC or LU variable (Figure 3). For the other data sets, both the mean NDVI and NDWI from the ancillary variables were important for Dar es Salaam and no LCZ covariate was found to be important for any of the four cities.

### 3.3 Predictive Maps

**Figure 4:**
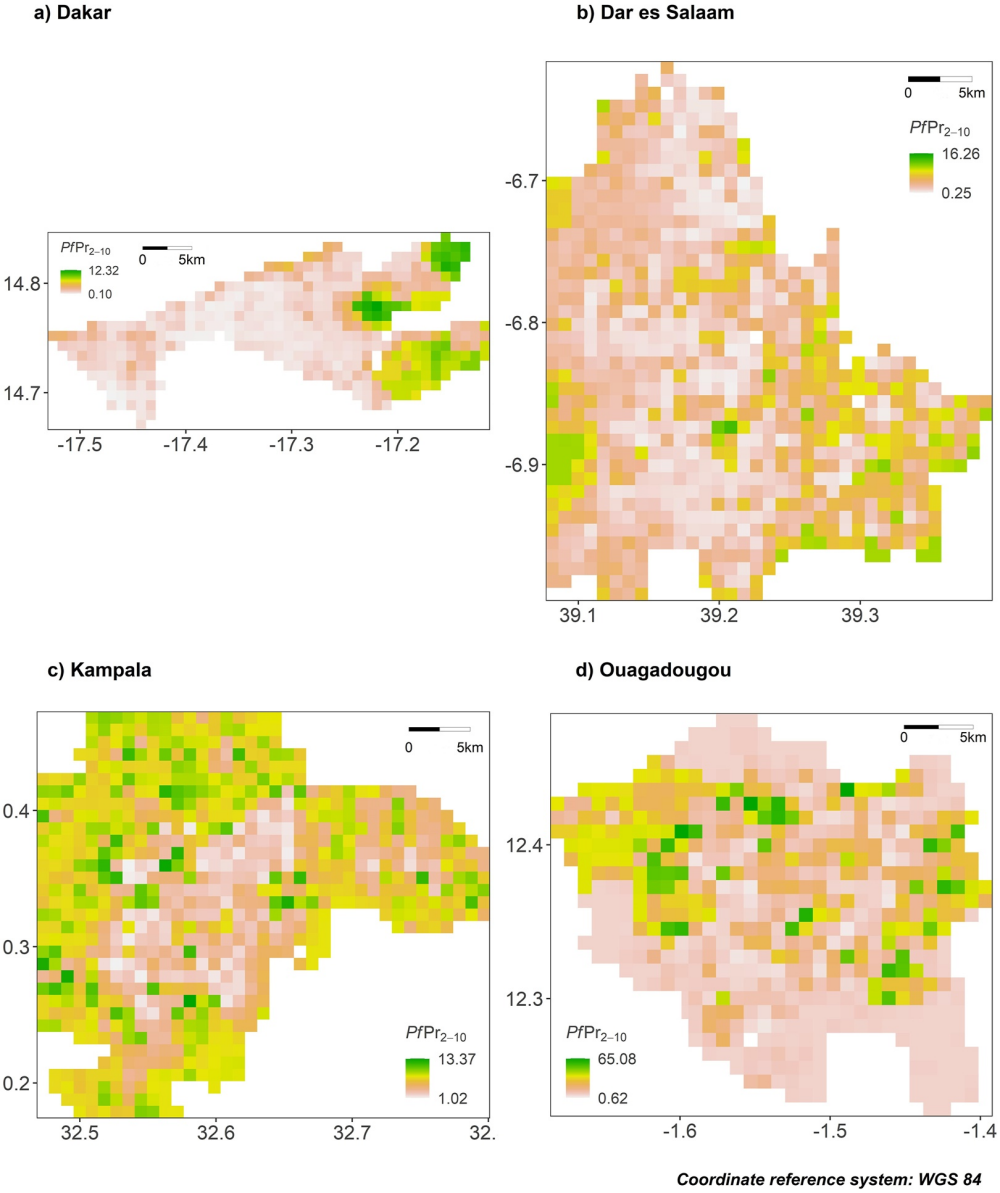
Predicted *Pf*PR_2–10_ at 1 km resolution for Dakar, Dar es Salaam, Kampala and Ouagadougou. *Pf*PR_2–10_ was predicted using the important covariates selected by recursive feature elimination and using the best performing method for each city, that is, M1 for Dakar and M2 for Dar es Salaam, Kampala and Ouagadougou. The final predicted values are obtained by averaging the predictions of 25 random forest models built in spatial cross‐validation.

Using the best models, we were able to predict *Pf*PR_2–10_ at 1 km resolution in the four cities of interest. Predictive maps revealed two different types of spatial trends. In Dakar and Kampala (Figures 4a and 4c), the predicted intra‐urban malaria risk increased from the city center to the peri‐urban areas. In Dar es Salaam and Ouagadougou (Figures 4b and 4d), however, the intra‐urban malaria risk did not follow this classical increasing trend from the city center to the outskirts, but developed as central hotspots where some specific environmental conditions (LCLU) were met. In Dar es Salaam in particular, hotspots developed around water channels.

### 3.4 Simulation Study

**Figure 5:**
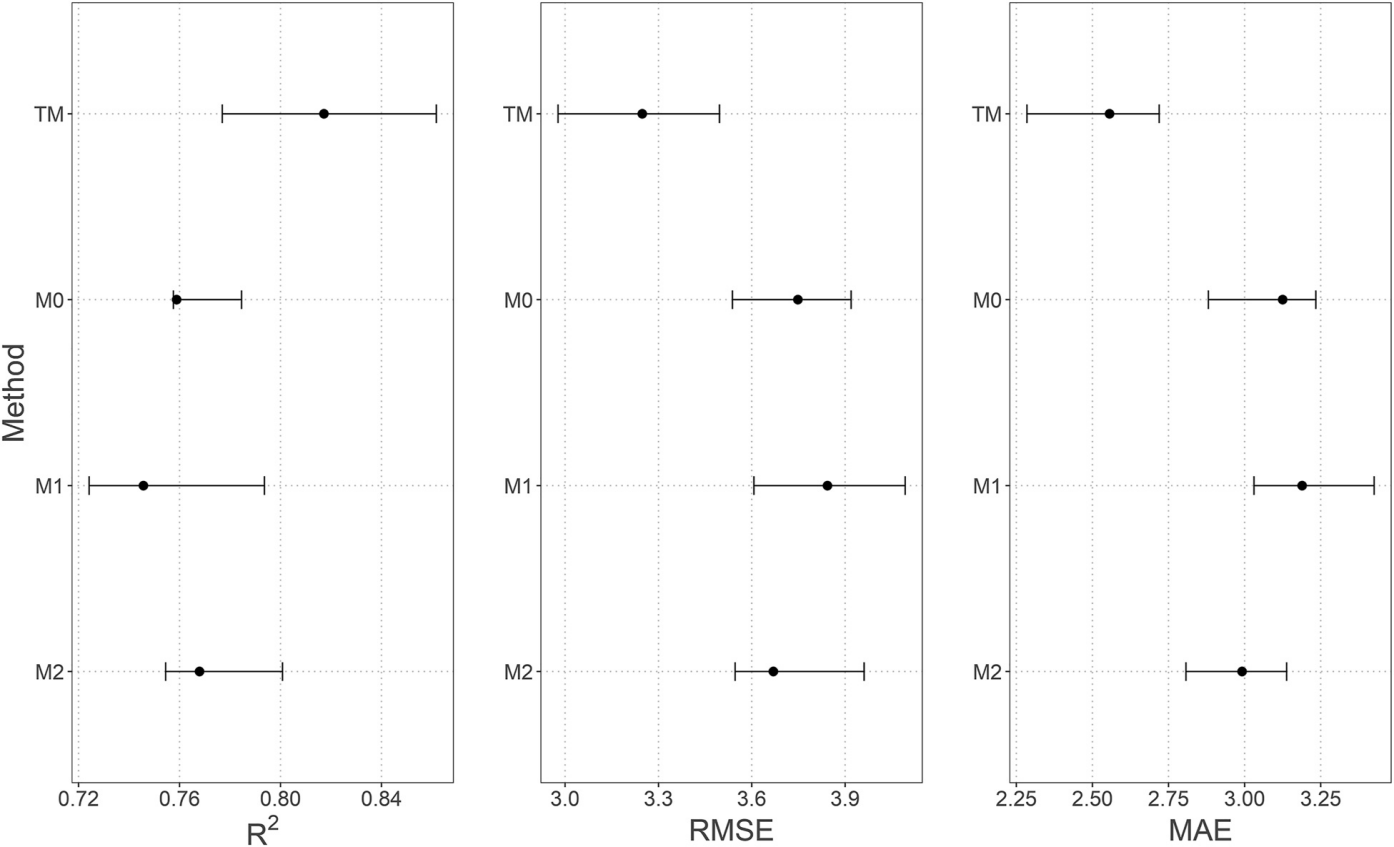
Average *R* ^2^, root mean square error (RMSE) and mean absolute error (MAE) scores obtained with true model (TM), M0, M1 and M2. The thick dots are the averaged metrics (*R* ^2^, RMSE and MAE) over the 10 repetitions of the simulation study and the error bars represent the interquartile range calculated over these repetitions.

Figure 5 and Table 4 show the predictive performance (RMSE, MAE and *R* ^2^) of M0, M1 and M2 applied to the simulated displaced data for Dar es Salaam, and of TM, the RF models trained on the original non‐displaced data. GoF indices were calculated after prediction on the non‐displaced sets and averaged over 10 iterations of the simulation study.

**Table 4:** Average *R* ^2^, Root Mean Square Error (RMSE) and Mean Absolute Error (MAE) Scores Obtained With True Model (TM), M0, M1 and M2, for the Simulation Study in Dar es Salaam

Method	*R* ^2^	RMSE	MAE
TM	0.82	3.25	2.56
M0	0.76	3.75	3.12
M1	0.75	3.84	3.19
M2	0.77	3.67	2.99

*Note*. The scores are averaged over 10 repetitions of the simulation study.

Randomly shifting the data points from their location following the DHS displacement procedure resulted, as expected, in a decrease in prediction performance in terms of all three GoF indices; the *R* ^2^ decreased from 0.82 (TM) to 0.76 (M0), the RMSE increased from 3.25 (TM) to 3.75 (M0), and the MAE increased from 2.56 (TM) to 3.12 (M0) (Table 4). M1 and M2 did not fully recover the performance of TM, but on average M2 slightly outperformed M0 in terms of all three GoFs, with an average *R* ^2^ of 0.77, an average RMSE of 3.67 and an average MAE of 2.99. In contrast, M1 did not improve the performance over M0 on any of the three GoF indices. These results are consistent with previous results from modeling malaria risk in Dar es Salaam using DHS, where M2 outperformed M0 but this was not the case for M1 (Table 3). Note that there are limitations associated with this simulation study that could affect the performance of the spatial optimization methods (see Discussion section).

## 4 Discussion

In the current context of climate change and rapid and unplanned urbanization in SSA, there is an urgent need to deepen the knowledge of intra‐urban malaria risk and to develop predictive maps for SSA cities, where malaria risk is known to be highly heterogeneous (Brown et al., 2020; Mathanga et al., 2016). However, such attempts have always been constrained by the availability and quality of malaria survey data, which, although improving considerably, remain limited for studying malaria risk at the intra‐urban scale. In this study, we tested the potential of spatial optimization methods to overcome the loss of spatial accuracy due to DHS cluster displacement in modeling and predicting intra‐urban malaria risk. For all cities, regardless of the proportion of DHS data points used to build the models, at least one of the two spatial optimization methods investigated in this study improved the performance of the RF models compared to models for which no spatial optimization method was used. However, the performance improvement was small, while using the spatial optimization methods increased the computational cost. We also tested the spatial optimization methods on non‐DHS data from Dar es Salaam, for which we simulated a DHS displacement. Randomly shifting the survey locations reduced the predictive performance of the models as expected, as shown in previous work (Burgert et al., 2013; Perez‐Heydrich et al., 2013). Only one of the two spatial optimization methods (M2) improved the predictive performance in the simulation. However, the predictive performance of the spatial optimization methods in the simulation study may be affected by the large uncertainty in the accuracy of the coordinates of the non‐DHS data used to run the simulation. While some of these surveys have precise GPS coordinates, others are based on the surveyor's best estimate of the location of the surveys, and still others are school surveys where the actual residence of the respondents is unknown (Macharia et al., 2022).

Overall, the GoF indices (*R* ^2^, RMSE and MAE) obtained with the spatial optimization methods for the four cities of interest are in line with previous studies that have modeled *Pf*PR_2–10_ at the intra‐urban scale without using DHS data but using similar covariates and modeling frameworks (Brousse et al., 2020b; Georganos et al., 2020; Morlighem et al., 2022). Rather than using only DHS or non‐DHS data separately, the inclusion of both types of surveys in the models can assist the spatial optimization methods in finding back plausible locations for the (displaced) DHS data points, while increasing the number of observations available to build the models. This is done by providing the models with some information about the relationships between the predictors and *Pf*PR_2–10_ derived from the non‐displaced observations. For example, if there is a positive relationship between *Pf*PR_2–10_ and the proportion of water in the non‐displaced data, and if this is found by the RF models, the spatial optimization method based on refining feature extraction (M2) might tend to select duplicates of DHS data points created at locations that support this relationship.

Nevertheless, the findings of this research should be used with caution, as our models explained only about 30%–40% of the variance of *Pf*PR_2–10_. In addition to the expected lower performance due to the use of spatial cross‐validation (Meyer et al., 2019), several limitations may explain this low percentage of explained variance. First, we assumed temporal stationarity of malaria risk, as malaria surveys are poorly distributed within our 10‐year period (2005–2015) and are not available in sufficient numbers to make seasonal or annual predictions. We also did not capture temporal variation in the covariates, as they were not collected at the same time either in relation to each other or in relation to the malaria surveys. Instead, where sufficient data were available, we aggregated covariates over a long time period (i.e., 10 years) to smooth out temporal variations influenced by exceptional weather conditions. In addition to assuming temporal stationarity, we also assumed a stationary population and ignored the effect of human mobility on malaria risk. Yet, human mobility plays an important role in the transmission of vector‐borne diseases, as population movements lead to continuous pathogen reintroductions (Buckee et al., 2013; Wesolowski et al., 2012). Future studies should investigate the use of mobile phone and social media data to recover human mobility patterns and test the added value of this information for malaria risk mapping. Our models also lacked information on human behavior related to malaria prevention and anticipation (e.g., use of bed nets, antimalarial drugs, or indoor residual spraying), which could also be the focus of future work.

Alongside those methodological limitations, there are also some limitations related to the epidemiological data available to conduct this research, and these are inherent to DHS. First, while MIS are generally conducted during the high transmission season, that is, the rainy season and subsequent weeks, this is not the case for DHS (Massoda Tonye et al., 2018). However, the *Pf*PR is highest during the wetter months and drops during the dry season. Seasonal variation in *Pf*PR also leads to seasonal variation in malaria transmission and human behavior, such as increased use of insecticide‐treated nets during the rainy season (Massoda Tonye et al., 2018; Ozodiegwu et al., 2021; Wright et al., 2012). This raises comparability issues between annual DHS conducted at different time periods, but also between DHS and MIS (Massoda Tonye et al., 2018; Ozodiegwu et al., 2021; Wright et al., 2012). Furthermore, as the DHS sampling frame is defined to provide indicators that are representative at the national level, the number of survey clusters at the intra‐urban level for a given DHS is often insufficient to model DHS indicators using data from that specific DHS only. Instead, mapping malaria risk at this scale requires combining several years of data collection, assuming stable malaria prevalence over the period, as was done in this study and in other previous work (Brousse et al., 2020b; Georganos et al., 2020; Kabaria et al., 2016; Morlighem et al., 2022). This limits the potential to analyze annual/seasonal trends in malaria risk at the intra‐urban scale, for example using spatio‐temporal models. Another challenge with DHS is that they typically measure malaria prevalence by testing children under the age of five, as malaria is known to be most prevalent in this age group (Smith et al., 2007), before standardizing to the 2 to 10 age range. However, the final *Pf*PR_2–10_ may be underestimated as antimalarial drug use is more prevalent in young children due to their lower immunity and therefore more severe clinical malaria reactions when infected (Smith et al., 2007). This is exacerbated  by the fact that DHS are conducted independently of national intervention programmes (Ozodiegwu et al., 2021). DHS are sometimes conducted during chemoprevention interventions that target young children and significantly reduce the *Pf*PR in this specific age group, while having less impact on other groups (Ozodiegwu et al., 2021). Finally, DHS sometimes have low spatial accuracy, partly due to instrumental biases (sampling errors in GPS receivers), but mainly due to the displacement of survey cluster coordinates by up to 2 km in urban extents for privacy protection (Burgert et al., 2013; Corsi et al., 2012; Georganos et al., 2019; Ozodiegwu et al., 2021).

To address these challenges, we highlight here potential adaptations to the DHS sampling strategy, as already encouraged by Ozodiegwu et al. (2021). Specifically, we propose to (a) systematically plan DHS during the high transmission season, that is, the rainy season, so that *Pf*PR estimates are comparable between surveys; (b) increase survey frequency where possible, especially in high‐risk areas; (c) include children up to 10 years of age in testing to avoid the potential underestimation of *Pf*PR_2–10_ when only younger children are tested; and (d) adapt the DHS strategy based on national control interventions to measure their impact and avoid intervention‐related bias. In addition, we propose to re‐think the geographical displacement procedure of DHS cluster centroids. This could be achieved by reducing the displacement distance in areas of high population density, at least in urban areas, where displacement has a greater impact on the analysis potential due to the high heterogeneity of the cityscape. Restrictions could also be applied to the survey coordinates, forcing them to stay within the city extents or within the land use or land cover class to which they belong. Urban masks based on layers such as GHSL (European Commission, 2022) or GUF (DLR, 2022) could be used to support this displacement. Following the same idea, the displacement could also be constrained by the boundaries of the lower administrative unit level, that is, the third administrative level instead of the second. Recently, the DHS program has started to investigate alternative displacement methods, revising its spatial Anonymisation approach by using population maps to calculate the minimum distance to which a cluster centroid must be displaced such that anonymity is preserved (The DHS Program & the Development Data Group of the World Bank, 2021). With further work in this direction and consideration of the proposed changes, DHS would become a valuable tool for mapping intra‐urban malaria risk, especially in malaria‐endemic countries where DHS are the only available source of epidemiological data.

Our findings also have future implications for malaria risk mapping in SSA cities where malaria data are too limited in both quantity and quality. As already suggested by Brousse et al. (2020b), when SSA cities share the same socioeconomic and environmental contexts, the extrapolation capacity of models calibrated on data‐rich cities may be tested to predict *Pf*PR_2–10_ in data‐poor cities. Future work will further investigate this extrapolation capacity and the potential for upscaling methods. Given the rapid and profound environmental changes in SSA cities that are likely to affect the burden of vector‐borne diseases in the future, risk mapping methods tailored to African urban areas are urgently needed to help target control interventions. We expect the quality and quantity of available data on malaria and other vector‐borne diseases to improve in the future, which will certainly make these methods extremely useful in the fight against these diseases.

## Conflict of Interest

The authors declare no conflicts of interest relevant to this study.

## Data Availability

The R scripts needed to perform the analyses are available from an open access Zenodo repository (Morlighem et al., 2023). The malaria prevalence data are freely available in the Harvard Dataverse repository (Snow et al., 2017a). All geographical variables used in this study are also freely available. LC and LU products are publicly available from Zenodo repositories (Georganos, 2020; Georganos & Grippa, 2020a, 2020b; Grippa & Georganos, 2018a, 2018b, 2018c, 2019). LCZ maps can be downloaded from the LCZ Generator (https://lcz-generator.rub.de/) (Demuzere et al., 2021). Climate data sets are also open source products, with WorldClim data available from https://www.worldclim.org/ (Fick & Hijmans, 2017). Finally, MODIS (Wan et al., 2015a, 2015b), SRTM and Landsat 5 and 8 imagery (from which we computed the NDVI and NDWI) can all be extracted from https://www.usgs.gov/.

## References

[gh2472-bib-0001] Adigun, A. B. , Gajere, E. N. , Oresanya, O. , & Vounatsou, P. (2015). Malaria risk in Nigeria: Bayesian geostatistical modelling of 2010 malaria indicator survey data. Malaria Journal, 14(1), 156. 10.1186/s12936-015-0683-6 25880096 PMC4404580

[gh2472-bib-0002] Boyce, M. R. , Katz, R. , & Standley, C. J. (2019). Risk factors for infectious diseases in urban environments of sub‐Saharan Africa: A systematic review and critical appraisal of evidence. Tropical Medicine and Infectious Disease, 4(4), 123. 10.3390/tropicalmed4040123 31569517 PMC6958454

[gh2472-bib-0003] Breiman, L. (2001). Random forests. Machine Learning, 45(1), 5–32. 10.1023/A:1010933404324

[gh2472-bib-0004] Brousse, O. , Georganos, S. , Bechtel, B. , Droste, A. , Thiery, W. , van Lipzig, N. P. , & Demuzere, M. (2020a). Mapping African cities in local climate zones: Reporting on a mapathon experience (Vol. 76). IAUC Newsletter. Retrieved from http://www.urban-climate.org/wp-content/uploads/newsletter/IAUC076.pdf

[gh2472-bib-0005] Brousse, O. , Georganos, S. , Demuzere, M. , Dujardin, S. , Lennert, M. , Linard, C. , et al. (2020b). Can we use local climate zones for predicting malaria prevalence across sub‐Saharan African cities? Environmental Research Letters, 15(12), 124051. 10.1088/1748-9326/abc996 35211191 PMC7612418

[gh2472-bib-0006] Brousse, O. , Thiery, W. , Demuzere, M. , & Lipzig, N. (2020c). Urban climates and their effect on malaria in sub‐Saharan Africa (PhD Thesis).

[gh2472-bib-0007] Brown, B. J. , Manescu, P. , Przybylski, A. A. , Caccioli, F. , Oyinloye, G. , Elmi, M. , et al. (2020). Data‐driven malaria prevalence prediction in large densely populated urban holoendemic sub‐Saharan West Africa. Scientific Reports, 10(1), 15918. 10.1038/s41598-020-72575-6 32985514 PMC7522256

[gh2472-bib-0008] Buckee, C. O. , Wesolowski, A. , Eagle, N. N. , Hansen, E. , & Snow, R. W. (2013). Mobile phones and malaria: Modeling human and parasite travel. Travel Medicine and Infectious Disease, 11(1), 15–22. 10.1016/j.tmaid.2012.12.003 23478045 PMC3697114

[gh2472-bib-0009] Burgert, C. R. , Colston, J. M. , Roy, T. , & Zachary, B. (2013). Geographic displacement procedure and georeferenced data release policy for the Demographic and Health Surveys. DHS Spatial Analysis Reports No. 7. ICF International.

[gh2472-bib-0010] Corsi, D. J. , Neuman, M. , Finlay, J. E. , & Subramanian, S. (2012). Demographic and health surveys: A profile. International Journal of Epidemiology, 41(6), 1602–1613. 10.1093/ije/dys184 23148108

[gh2472-bib-0011] Demuzere, M. , Kittner, J. , & Bechtel, B. (2021). LCZ generator: A web application to create local climate zone maps. Frontiers in Environmental Science, 9(112), 637455. 10.3389/fenvs.2021.637455

[gh2472-bib-0012] DLR . (2022). DLR ‐ Earth Observation Center ‐ Global urban footprint. Retrieved from https://www.dlr.de/eoc/en/desktopdefault.aspx/tabid-9628/16557_read-40454/

[gh2472-bib-0013] Doumbe‐Belisse, P. , Kopya, E. , Ngadjeu, C. S. , Sonhafouo‐Chiana, N. , Talipouo, A. , Djamouko‐Djonkam, L. , et al. (2021). Urban malaria in sub‐Saharan Africa: Dynamic of the vectorial system and the entomological inoculation rate. Malaria Journal, 20(1), 364. 10.1186/s12936-021-03891-z 34493280 PMC8424958

[gh2472-bib-0014] Ejigu, B. A. (2020). Geostatistical analysis and mapping of malaria risk in children of Mozambique. PLoS One, 15(11), e0241680. 10.1371/journal.pone.0241680 33166322 PMC7652261

[gh2472-bib-0015] Esri , DigitalGlobe , GeoEye , i‐cubed , USDA FSA , USGS , et al. (2021). World imagery [Basemap]. Retrieved from https://www.arcgis.com/home/item.html?id=10df2279f9684e4a9f6a7f08febac2a9

[gh2472-bib-0016] European Commission . (2022). Global human settlement ‐ GHSL homepage ‐ European Commission. Retrieved from https://ghsl.jrc.ec.europa.eu/

[gh2472-bib-0017] Fick, S. E. , & Hijmans, R. J. (2017). WorldClim 2: New 1‐km spatial resolution climate surfaces for global land areas. International Journal of Climatology, 37(12), 4302–4315. 10.1002/joc.5086

[gh2472-bib-0018] Georganos, S. (2020). Malaria in high‐resolution: Modelling and mapping plasmodium falciparum parasite rate using very‐high‐resolution satellite derived indicators in sub‐Saharan African cities [Dataset]. Zenodo. 10.5281/zenodo.3871497 PMC7504835 32958055

[gh2472-bib-0019] Georganos, S. , Brousse, O. , Dujardin, S. , Linard, C. , Casey, D. , Milliones, M. , et al. (2020). Modelling and mapping the intra‐urban spatial distribution of plasmodium falciparum parasite rate using very‐high‐resolution satellite derived indicators. International Journal of Health Geographics, 19(1), 38. 10.1186/s12942-020-00232-2 32958055 PMC7504835

[gh2472-bib-0020] Georganos, S. , Gadiaga, A. N. , Linard, C. , Grippa, T. , Vanhuysse, S. , Mboga, N. , et al. (2019). Modelling the wealth index of demographic and health surveys within cities using very high‐resolution remotely sensed information. Remote Sensing, 11(21), 2543. 10.3390/rs11212543

[gh2472-bib-0021] Georganos, S. , & Grippa, T. (2020a). Dar es Salaam very‐high‐resolution land cover map [Dataset]. Zenodo. 10.5281/zenodo.3711903

[gh2472-bib-0022] Georganos, S. , & Grippa, T. (2020b). Kampala very‐high‐resolution land cover map [Dataset]. Zenodo. 10.5281/zenodo.3711905

[gh2472-bib-0023] Gething, P. W. , Tatem, A. J. , Bird, T. J. , & Burgert‐Brucker, C. R. (2015). Creating spatial interpolation surfaces with DHS data. DHS Spatial Analysis Reports No. 11. ICF International.

[gh2472-bib-0024] Giardina, F. , Gosoniu, L. , Konate, L. , Diouf, M. B. , Perry, R. , Gaye, O. , et al. (2012). Estimating the burden of malaria in Senegal: Bayesian zero‐inflated binomial geostatistical modeling of the MIS 2008 data. PLoS One, 7(3), e32625. 10.1371/journal.pone.0032625 22403684 PMC3293829

[gh2472-bib-0025] Gregorutti, B. , Michel, B. , & Saint‐Pierre, P. (2017). Correlation and variable importance in random forests. Statistics and Computing, 27(3), 659–678. 10.1007/s11222-016-9646-1

[gh2472-bib-0026] Grippa, T. , & Georganos, S. (2018a). Dakar land use map at street block level (V1.0) [Dataset]. Zenodo. 10.5281/zenodo.1291389

[gh2472-bib-0027] Grippa, T. , & Georganos, S. (2018b). Dakar very‐high resolution land cover map (V1.0) [Dataset]. Zenodo. 10.5281/zenodo.1290800

[gh2472-bib-0028] Grippa, T. , & Georganos, S. (2018c). Ouagadougou very‐high resolution land cover map (V1.0) [Dataset]. Zenodo. 10.5281/zenodo.1290654

[gh2472-bib-0029] Grippa, T. , & Georganos, S. (2019). Ouagadougou land use map at street block level (V2.0) [Dataset]. Zenodo. 10.5281/zenodo.3238302

[gh2472-bib-0030] Grippa, T. , Georganos, S. , Zarougui, S. , Bognounou, P. , Diboulo, E. , Forget, Y. , et al. (2018). Mapping urban land use at street block level using OpenStreetMap, remote sensing data, and spatial metrics. International Journal of Geo‐Information, 7(7), 246. 10.3390/ijgi7070246

[gh2472-bib-0031] Grippa, T. , Lennert, M. , Beaumont, B. , Vanhuysse, S. , Stephenne, N. , & Wolff, E. (2017). An open‐source semi‐automated processing chain for urban object‐based classification. Remote Sensing, 9(4), 358. 10.3390/rs9040358

[gh2472-bib-0032] Hay, S. I. , & Snow, R. W. (2006). The malaria atlas project: Developing global maps of malaria risk. PLoS Medicine, 3(12), e473. 10.1371/journal.pmed.0030473 17147467 PMC1762059

[gh2472-bib-0033] Kabaria, C. W. , Molteni, F. , Mandike, R. , Chacky, F. , Noor, A. M. , Snow, R. W. , & Linard, C. (2016). Mapping intra‐urban malaria risk using high resolution satellite imagery: A case study of Dar es Salaam. International Journal of Health Geographics, 15(1), 26. 10.1186/s12942-016-0051-y 27473186 PMC4967308

[gh2472-bib-0034] Lovelace, R. , Nowosad, J. , & Muenchow, J. (2019). Statistical learning. In Geocomputation with R. Boca Raton. Chapman and Hall/CRC.

[gh2472-bib-0035] Macharia, P. M. , Ray, N. , Gitonga, C. W. , Snow, R. W. , & Giorgi, E. (2022). Combining school‐catchment area models with geostatistical models for analysing school survey data from low‐resource settings: Inferential benefits and limitations. Spatial Statistics, 51, 100679. 10.1016/j.spasta.2022.100679 35880005 PMC7613137

[gh2472-bib-0036] Machault, V. , Vignolles, C. , Pages, F. , Gadiaga, L. , Gaye, A. , Sokhna, C. , et al. (2010). Spatial heterogeneity and temporal evolution of malaria transmission risk in Dakar, Senegal, according to remotely sensed environmental data. Malaria Journal, 9(1), 252. 10.1186/1475-2875-9-252 20815867 PMC2944340

[gh2472-bib-0037] Malaria Atlas Project . (2022). Welcome to the malaria atlas project ‐ MAP. Retrieved from https://malariaatlas.org/

[gh2472-bib-0038] Massoda Tonye, S. G. , Kouambeng, C. , Wounang, R. , & Vounatsou, P. (2018). Challenges of DHS and MIS to capture the entire pattern of malaria parasite risk and intervention effects in countries with different ecological zones: The case of Cameroon. Malaria Journal, 17(1), 156. 10.1186/s12936-018-2284-7 29625574 PMC5889563

[gh2472-bib-0039] Mathanga, D. P. , Tembo, A. K. , Mzilahowa, T. , Bauleni, A. , Mtimaukenena, K. , Taylor, T. E. , et al. (2016). Patterns and determinants of malaria risk in urban and peri‐urban areas of Blantyre, Malawi. Malaria Journal, 15(1), 590. 10.1186/s12936-016-1623-9 27931234 PMC5146950

[gh2472-bib-0040] Meyer, H. , Reudenbach, C. , Wöllauer, S. , & Nauss, T. (2019). Importance of spatial predictor variable selection in machine learning applications – Moving from data reproduction to spatial prediction. Ecological Modelling, 411, 108815. 10.1016/j.ecolmodel.2019.108815

[gh2472-bib-0041] Morlighem, C. , Chaiban, C. , Georganos, S. , Brousse, O. , Van de Walle, J. , van Lipzig, N. P. M. , et al. (2022). The multi‐satellite environmental and socioeconomic predictors of vector‐borne diseases in African cities: Malaria as an example. Remote Sensing, 14(21), 5381. 10.3390/rs14215381

[gh2472-bib-0042] Morlighem, C. , Chaiban, C. , Georganos, S. , Brousse, O. , van Lipzig, N. P. M. , Wolff, E. , et al. (2023). Mapping malaria risk in sub‐Saharan African cities [Dataset]. Zenodo. 10.5281/zenodo.8082712 PMC10558065 37811342

[gh2472-bib-0043] Ozodiegwu, I. D. , Ambrose, M. , Battle, K. E. , Bever, C. , Diallo, O. , Galatas, B. , et al. (2021). Beyond national indicators: Adapting the demographic and health surveys' sampling strategies and questions to better inform subnational malaria intervention policy. Malaria Journal, 20(1), 122. 10.1186/s12936-021-03646-w 33648499 PMC7919087

[gh2472-bib-0044] Perez‐Heydrich, C. , Warren, J. , Burgert, C. , & Emch, M. (2013). Guidelines on the use of DHS GPS data. Spatial Analysis Reports No. 8. ICF International.

[gh2472-bib-0045] Riedel, N. , Vounatsou, P. , Miller, J. M. , Gosoniu, L. , Chizema‐Kawesha, E. , Mukonka, V. , & Steketee, R. W. (2010). Geographical patterns and predictors of malaria risk in Zambia: Bayesian geostatistical modelling of the 2006 Zambia national malaria indicator survey (ZMIS). Malaria Journal, 9(1), 37. 10.1186/1475-2875-9-37 20122148 PMC2845589

[gh2472-bib-0046] Robert, V. , Macintyre, K. , Keating, J. , Trape, J. F. , Duchemin, J. B. , Warren, M. , & Beier, J. C. (2003). Malaria transmission in urban sub‐Saharan Africa. The American Journal of Tropical Medicine and Hygiene, 68(2), 169–176. 10.4269/ajtmh.2003.68.169 12641407

[gh2472-bib-0047] Rodríguez, E. , Morris, C. S. , & Belz, J. E. (2006). A global assessment of the SRTM performance. Photogrammetric Engineering & Remote Sensing, 72(3), 249–260. 10.14358/PERS.72.3.249

[gh2472-bib-0048] Rogers, D. J. , Randolph, S. E. , Snow, R. W. , & Hay, S. I. (2002). Satellite imagery in the study and forecast of malaria. Nature, 415(6872), 710–715. 10.1038/415710a 11832960 PMC3160466

[gh2472-bib-0049] Serdeczny, O. , Adams, S. , Baarsch, F. , Coumou, D. , Robinson, A. , Hare, W. , et al. (2017). Climate change impacts in sub‐Saharan Africa: From physical changes to their social repercussions. Regional Environmental Change, 17(6), 1585–1600. 10.1007/s10113-015-0910-2

[gh2472-bib-0050] Sewe, M. O. , Ahlm, C. , & Rocklöv, J. (2016). Remotely sensed environmental conditions and malaria mortality in three malaria endemic regions in Western Kenya. PLoS One, 11(4), e0154204. 10.1371/journal.pone.0154204 27115874 PMC4845989

[gh2472-bib-0051] Sinka, M. E. , Bangs, M. J. , Manguin, S. , Rubio‐Palis, Y. , Chareonviriyaphap, T. , Coetzee, M. , et al. (2012). A global map of dominant malaria vectors. Parasites & Vectors, 5(1), 69. 10.1186/1756-3305-5-69 22475528 PMC3349467

[gh2472-bib-0052] Smith, D. L. , Guerra, C. A. , Snow, R. W. , & Hay, S. I. (2007). Standardizing estimates of the plasmodium falciparum parasite rate. Malaria Journal, 6(1), 131. 10.1186/1475-2875-6-131 17894879 PMC2072953

[gh2472-bib-0053] Snow, R. W. , Sartorius, B. , Kyalo, D. , Maina, J. , Amratia, P. , Mundia, C. W. , et al. (2017a). The prevalence of plasmodium falciparum in sub Saharan Africa since 1900 [Dataset]. Harvard Dataverse, 550(7677), 515–518. 10.7910/DVN/Z29FR0 PMC5660624 29019978

[gh2472-bib-0054] Snow, R. W. , Sartorius, B. , Kyalo, D. , Maina, J. , Amratia, P. , Mundia, C. W. , et al. (2017b). The prevalence of plasmodium falciparum in sub‐Saharan Africa since 1900. Nature, 550(7677), 515–518. 10.1038/nature24059 29019978 PMC5660624

[gh2472-bib-0055] Ssempiira, J. , Nambuusi, B. , Kissa, J. , Agaba, B. , Makumbi, F. , Kasasa, S. , & Vounatsou, P. (2017). Geostatistical modelling of malaria indicator survey data to assess the effects of interventions on the geographical distribution of malaria prevalence in children less than 5 years in Uganda. PLoS One, 12(4), e0174948. 10.1371/journal.pone.0174948 28376112 PMC5380319

[gh2472-bib-0056] Tatem, A. J. , Gething, P. W. , Smith, D. L. , & Hay, S. I. (2013). Urbanization and the global malaria recession. Malaria Journal, 12(1), 133. 10.1186/1475-2875-12-133 23594701 PMC3639825

[gh2472-bib-0057] The DHS Program , & the Development Data Group of the World Bank . (2021). Spatial anonymization: Guidance note prepared for the inter‐Secretariat working group on household surveys.

[gh2472-bib-0058] The DHS Program . (2022). The DHS program ‐ Quality information to plan, monitor and improve population, health, and nutrition programs. Retrieved from https://dhsprogram.com/

[gh2472-bib-0059] United Nations , Department of Economic and Social Affairs , & Population Division . (2019). World urbanization prospects: The 2018 revision (ST/ESA/SER.A/420). United Nations.

[gh2472-bib-0060] Wan, Z. , Hook, S. , & Hulley, G. (2015a). MOD11A1 MODIS/terra land Surface temperature/emissivity daily L3 global 1km SIN grid V006 [Dataset]. NASA EOSDIS Land Processes DAAC. 10.5067/MODIS/MOD11A1.006

[gh2472-bib-0061] Wan, Z. , Hook, S. , & Hulley, G. (2015b). MYD11A2 MODIS/aqua land Surface temperature/emissivity 8‐day L3 global 1km SIN grid V006 [Dataset]. NASA EOSDIS Land Processes DAAC. 10.5067/MODIS/MYD11A2.006

[gh2472-bib-0062] Wesolowski, A. , Eagle, N. , Tatem, A. J. , Smith, D. L. , Noor, A. M. , Snow, R. W. , & Buckee, C. O. (2012). Quantifying the impact of human mobility on malaria. Science, 338(6104), 267–270. 10.1126/science.1223467 23066082 PMC3675794

[gh2472-bib-0063] World Health Organization . (2021). World malaria report 2021. World Health Organization.

[gh2472-bib-0064] Wright, J. , Yang, H. , & Walker, K. (2012). Do international surveys and censuses exhibit ‘Dry Season’ bias? Population, Space and Place, 18(1), 116–126. 10.1002/psp.681

